# On the Rapid Calculation of Binding Affinities for Antigen and Antibody Design and Affinity Maturation Simulations

**DOI:** 10.3390/antib11030051

**Published:** 2022-08-03

**Authors:** Simone Conti, Edmond Y. Lau, Victor Ovchinnikov

**Affiliations:** 1 Department of Chemistry and Chemical Biology, Harvard University, Cambridge, MA 02138, USA; 2 Physical and Life Sciences Directorate, Lawrence Livermore National Laboratory, Livermore, CA 94551, USA

**Keywords:** molecular dynamics simulation, free energy, protein-protein interaction, scoring function, protein-protein docking

## Abstract

The accurate and efficient calculation of protein-protein binding affinities is an essential component in antibody and antigen design and optimization, and in computer modeling of antibody affinity maturation. Such calculations remain challenging despite advances in computer hardware and algorithms, primarily because proteins are flexible molecules, and thus, require explicit or implicit incorporation of multiple conformational states into the computational procedure. The astronomical size of the amino acid sequence space further compounds the challenge by requiring predictions to be computed within a short time so that many sequence variants can be tested. In this study, we compare three classes of methods for antibody/antigen (Ab/Ag) binding affinity calculations: (i) a method that relies on the physical separation of the Ab/Ag complex in equilibrium molecular dynamics (MD) simulations, (ii) a collection of 18 scoring functions that act on an ensemble of structures created using homology modeling software, and (iii) methods based on the molecular mechanics-generalized Born surface area (MM-GBSA) energy decomposition, in which the individual contributions of the energy terms are scaled to optimize agreement with the experiment. When applied to a set of 49 antibody mutations in two Ab/HIV gp120 complexes, all of the methods are found to have modest accuracy, with the highest Pearson correlations reaching about 0.6. In particular, the most computationally intensive method, i.e., MD simulation, did not outperform several scoring functions. The optimized energy decomposition methods provided marginally higher accuracy, but at the expense of requiring experimental data for parametrization. Within each method class, we examined the effect of the number of independent computational replicates, i.e., modeled structures or reinitialized MD simulations, on the prediction accuracy. We suggest using about ten modeled structures for scoring methods, and about five simulation replicates for MD simulations as a rule of thumb for obtaining reasonable convergence. We anticipate that our study will be a useful resource for practitioners working to incorporate binding affinity calculations within their protein design and optimization process.

## 1. Introduction

In computational antibody design, the essential goal is to optimize an antibody sequence to increase the binding affinity to an antigen, subject to other biological constraints, such as solubility or lack of self-reactivity. Conversely, in computational vaccine design, a desired outcome of optimization is to create an antigen with a high affinity for a particular germline precursor, which can aid in the elicitation of a particular antibody lineage, e.g., to ensure a durable vaccine. Because the space of possible sequence variations is usually very large, even if only a few residue positions are allowed to vary, it is important to have a computational method that can make accurate and rapid predictions of antibody/antigen (Ab/Ag) binding free energy (bFE) changes upon sequence mutation.

Here, we report the results of our comparison of several methods of computing bFE, which vary in computational cost and accuracy (see Ref. [1] for a review of the underlying theory). At one end of the computational spectrum are physics-based methods that rely on all-atom classical molecular dynamics (MD) simulations to generate a thermodynamic ensemble of Ab/Ag structures in explicit solvent. These methods have the advantage that the only assumption is that the chosen classical potential energy function [2,3,4,5,6] provides an accurate approximation of the energy of the inherently quantum-mechanical, solvated protein complex. However, even using classical dynamics, such methods usually require very long simulation times, often hundreds of nanoseconds to microseconds [7] corresponding to billions to trillions of energy gradient evaluations, and thus, have not yet found routine use in practical antibody or antigen design. However, advances in simulation software that use graphical processor units (GPUs) [8,9,10,11] and in theoretical algorithms based on extended statistical mechanical ensembles [12,13] are continually making MD-based methods more competitive [7,14].

At the other end of the computational spectrum are more empirical methods, which use a scoring function to approximate bFEs directly, i.e., without relying on a thermodynamic ensemble of structures. Such methods technically only require a single structure, which can be obtained from the Protein Data Bank (PDB) [15], homology [16,17] or ab initio [18,19] modeling. However, because proteins are flexible in solution [20,21], the bound, unbound, and even transition states in the binding reaction [22] are composed of many different structures or microstates and macrostates [23,24] that contribute to binding. Thus, single-structure methods must implicitly represent potentially heterogeneous structural ensembles, which can limit their predictive accuracy [25].

An obvious idea to improve the accuracy of single-structure methods is to score multiple structures if they are available, e.g., from experiments [26], or from homology modeling software [16,18]. However, it is not generally clear how many structures should be used to achieve a good speed vs. accuracy compromise [27]. For example, different models produced by homology modeling programs can be structurally similar [16]. Further, sophisticated structure modeling algorithms can take minutes to hours [18], limiting high-throughput applications. When using thermodynamic ensemble-based methods such as MD, conformational landscape exploration can be very slow, because proteins evolve on rugged landscapes, trapped by large energy barriers [28,29,30]. Therefore, even for these methods it may be advantageous to repeat the simulations starting from different initial structures, or from different velocity realizations. Thus, aside from evaluating different methods for computing Ab/Ag binding affinity, we investigate the effect of the number of structures on the accuracy of the computed affinities. Single-structure scoring methods, as well as MD simulations, are tested with multiple structures or simulation replicates, respectively.

## 2. Materials and Methods

We explored three types of approaches for computing protein-protein binding affinities. To establish an approximate baseline for the expected accuracy of our calculations, we began with a computationally intensive approach based on molecular dynamics (MD) simulations, because an MD-based method that used alchemical free energy perturbation (FEP) previously achieved very good agreement with experiments [7]. To reduce computational cost, we next tested a variety of fast scoring functions developed for docking, protein folding, or protein–ligand binding. Finally, we tested several implicit solvent models, using an energy decomposition similar to that used in molecular mechanics-generalized Born surface area (MM-GBSA) models, over ensembles of up to twenty model structures. In this section, we first describe the experimental dataset used to test the calculations. Then, the three types of approaches are described in turn. We conclude this section with a description of the correlation analysis used to compare the computational results with the experimental data.

### 2.1. Experimental HIV Antibody–Antigen Binding Affinities

Because our primary interests are antibody and antigen design, and affinity maturation simulations for modeling HIV [31], as the experimental dataset we chose binding affinities between the HIV envelope glycoprotein (gp120) and the VRC01 broadly neutralizing antibody (bnAb), obtained from two studies. First, Clark et al. [32] published a dataset of 86 binding affinities for three different antibody–antigen complexes. They used the resurfaced stabilized core 3 (RSC3) antigen bound to antibodies VRC01, VRC03, or VRC-PG04. Because of the high overall computational expense of this study, here we only considered the 30 binding affinities relative to VRC01: the unmutated complex and 29 single-point alanine mutants of the antibody. The average experimental error for this dataset was estimated by the authors at 0.45 kcal/mol [7]. To model the structures of the antibody/antigen (Ab/Ag) complexes, the PDB structure 3NGB [32] was used as the template: it contains the correct antibody, but the antigen is 93TH057 instead of RSC3; unfortunately, no structure for RSC3 is available in the PDB. The main difference between the two antigens is the absence of a linker in RSC3, between residues 75 and 88. Following Clark et al. [7], we did not model the linker; instead, we split the protein chains into two parts. The second set of experimental binding affinities was taken from the study by Zhou et al. [32], who studied the effect of mutations for the complex between 93TH057 and VRC01. Experimental binding affinities were available for the unmutated complex, 12 single-point mutations, 3 revertants (4, 7, and 11 mutations each), 1 double mutation, and 2 insertions (AA and SY), for a total of 19 values. Structure 3NGB [32] was again used as the template, which matched both the antibody and the antigen used in the experiments. Thus, a total of 49 experimental binding affinities were obtained from the two sources (see Table 1). In all of the modeled complexes, only the variable part of the antibody was included; the constant region was deleted by cutting the heavy and light chains before the patterns SS[AP]ST and [KHR]RT, respectively. Residues 1 and 2 of the VRC01 light chain were not built as they were missing in the 3NGB structure.

### 2.2. Binding Affinities from Potentials of Mean Force (PMF) Simulations

Among the most accurate and physically motivated methods for simulating biomolecules are classical molecular dynamics simulations [33], which use carefully parametrized Newtonian energy functions (force fields) to approximate the motions of individual atoms [2], often immersed in explicit solvent molecules. Using classical statistical thermodynamics [34,35], it is possible to compute, essentially from first principles, the equilibrium free energies of the binding between proteins and ligands using restraining potentials [36,37] or alchemical transformations [38]. Because of the requirement of computational efficiency, here we used a fast method developed by us [39], in which the molecular solvent is modeled as a thin flexible layer (typically 7–10 Å in thickness) of explicit solvent molecules around the biomolecules of interest, with the bulk solvent omitted. When this method was applied to compute the potential of mean force (PMF) of separating an influenza hemagglutinin (HA) antigen from the bound anti-HA antibody [40], we observed very good agreement between the flexible-shell simulation results and those using a box filled with explicit water [39]. Thus, we also used the method in this study, with the approximations described below. As carried out by Clark et al. [7], we obtained coordinates for the antibody (Ab) VRC01 bound to an HIV-1 gp120 glycoprotein antigen (Ag) from the PDB file 3NGB [32]. We did not model HIV glycans, because they are generally not in close proximity to Ab loops. One exception is glycan NAG776, which was found previously to significantly affect the free energy of mutating residue Y28 in the VRC01 light chain to alanine [7]. However, our average computed ΔG (1.97 kcal/mol) was within 1 kcal/mol of the experiment (0.97 kcal/mol), which was lower than the combined RMS error between our simulations and experiment (about 2 kcal/mol). Thus, including this glycan would not likely improve our results.

Ab light and heavy chains were truncated to retain only the variable segments. Only residues 45–491 of the gp120 Ag were retained to reduce the system size. Although the HIV-1 spike is trimeric, only one Ag monomer bound to a single variable part of the antigen-binding antibody fragment (Fab) were simulated to reduce simulation cost. To prevent possible unphysical conformational changes due to the absence of stabilizing intermonomer contacts, weak RMS restraints were applied to the Ag with a force constant of 10 kcal/mol/Å^2^, which also prevented the overall translation and rotation of the Ag. Missing coordinates for loop residues 318–323 of gp120 were constructed using Modeller [41], and residue mutations to alanines were preformed using CHARMM [42] by truncating the side chains to the C_β_ atom (for glycines, by replacing the side-chain hydrogen with a methyl group). Due to the high computational expense of the MD-based PMF simulations, as described below, we only simulated the 30 alanine mutants (i.e., the VRC01/RSC03 dataset), as was performed by Clark et al. [7]. However, this reduced set was sufficient to establish that the PMF method did not outperform the less intensive scoring function methods (see Results).

To assign protonation states of ionizable residues, we checked the structures with the program ProPka3 [43]. Residues D, E, K, R, C, and Y were predicted to be in their standard protonation states. All histidines were predicted to be uncharged; the two histidines with the highest pKas were H72 and H85 in the gp120 subunit (pKa~6.6, i.e., close to neutral pH); however, they are both solvent-exposed and located about 30 angstroms from the binding interface, so that a change in their protonation state would not likely affect binding affinities. The histidine closest to the binding interface was ~11 angstroms away, with a pKa predicted at ~6.1. Because all of the histidines were relatively far away from the binding interface, the location of the proton (Nd vs. Ne) is unlikely to affect the binding affinity, and thus, the proton was placed on Nd.

Ag/Ab complexes were immersed in a large box of water solvent, water molecules beyond 7.5 Å were removed, and Na^+^ and Cl^−^ ions were added, as needed, to neutralize the systems and to bring total ionic concentration to approximately 0.15 M. The total atom counts for the solvated systems were around 19,000 atoms; they were not identical because of the residue mutations.

MD simulations were performed using the CHARMM36 energy function [2] and PME long-range electrostatics [44] using the program OpenMM [9,45], with the periodic box size set to 128 Å in each dimension. Lennard-Jones interactions were smoothly attenuated to zero in the range 7.5 Å–9 Å using the OpenMM cutoff function. Hydrogen masses were increased to 4 a.m.u. by transferring mass from the corresponding bound heavy atom, which permitted the use of a 4fs time step. To maintain the temperature at 300 K, we used the Langevin dynamics thermostat coupled to all atoms with a friction of γ = 0.1 ps.

**Figure 1:**
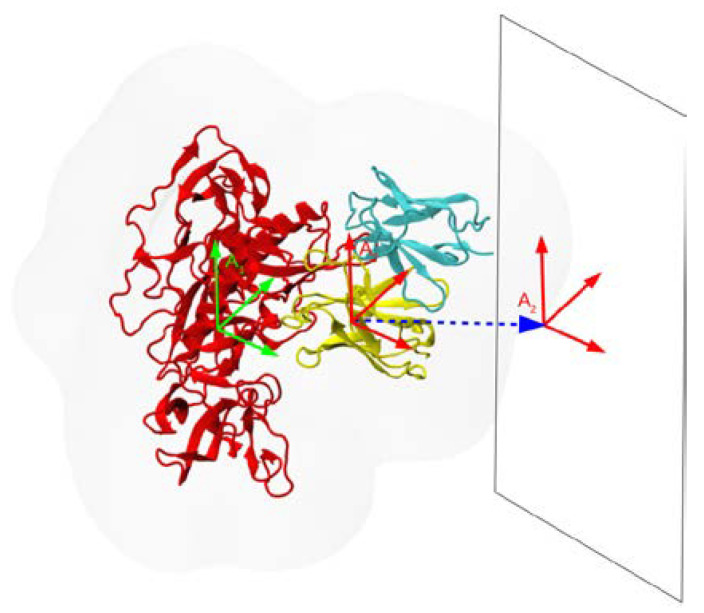
Schematic of the procedure used to compute PMFs of dissociation of Ab from the HIV gp120 Ag. Starting from the bound Ab/Ag complex, the Ab is displaced away from the Ag (red) along the vector (blue dashes) that points from the COM of the Ab in the direction that separates the Ag and the Ab (heavy chain is in yellow, light chain in cyan). Only the initial position of the Ab is shown. To preserve the relative orientations of Ag and Ab (indicated by the coordinate axes A_1_ and A_2_), weak harmonic restraints on the RMSDs of the Ag and Ab are applied, as described in the text. The rectangle perpendicular to the reaction coordinate indicates the orientation of the additional flat-bottom restraints to prevent excessive in-plane motion of the Ab, as described in the text. The faint gray outline indicates the solvent shell.

To compute the potential of mean force (PMF) of separating the Ab from the Ag, we used the flat-bottom restraint method [46] implemented as an OpenMM plugin with the following parameters. The reaction coordinate (RC) was the distance along the vector connecting the centers of mass (COM) of the Ab and Ag. Eight flat-bottom windows were used, with a force constant of 100 kcal/mol/Å^2^. The windows were centered on the COM separation distances of 34.83, 35.60, 36.61, 37.72, 38.92, 40.17, 41.48, and 42.83 Å; the flat-bottom region of each window was 0.5 Å wide. To accelerate convergence of the PMF, a distance restraint was applied to the Ab in planes orthogonal to the RC. The orthogonal distance force constant was 100 kcal/mol/Å^2^, and a flat-bottom region of 2.5 Å was used; within this distance of the RC axis, translation was unhindered. In addition, Ab rotation was penalized using translation-invariant RMSD restraints with the force constant set to 10 kcal/mol/Å^2^. An illustration of the procedure is given in Figure 1.

The initial configuration in the first window, i.e., that with the shortest Ag/Ab separation distance, which corresponds to the bound complex, was taken from the PDB structure 3NGB. The initial configurations for each subsequent separation distance were generated by pulling apart the complex to the new RC value over 400 ps. The simulation duration was 180 ns per window. Prior to the analysis to compute PMFs, the first 45 ns of simulation data in each window was discarded as part of equilibration. Each simulation was repeated seven times with different random seeds to generate statistics. For each mutation, the bFE difference was computed by subtracting the value in the last window of the PMF corresponding to the mutated Ab from that corresponding to the PMF from the unmutated complex. MD simulations were performed on GPU-accelerated supercomputers at Oak Ridge, Lawrence Livermore, and Lawrence Berkeley National Laboratories; the simulation speed per window was about 100 ns/day.

We note that a rigorous calculation of absolute binding free energies typically involves a decomposition into conformational and rigid-body components [36,37,47], in which the second category is of entropic origin (i.e., the logarithm of the configurational space expansion factor due to translation and rotation). In our protocol, we did not explicitly compute the rotational contribution to the FE because we expected it to approximately be canceled out in the FE differences. We also did not account for the influence of weak conformational RMSD restraints on the complex, because we, likewise, assumed that their contributions would be canceled out in the FE differences. The second approximation was justifiable here because we only considered simple single-residue mutations to alanine, which were not expected to cause major conformational rearrangements of the Ab complementarity-determining regions (CDRs). Ideally, the FE contributions of conformational restraints should be removed reversibly [37]; however, this would render the estimation of FE differences too computationally costly to use in affinity maturation simulations, or in high-throughput design projects.

### 2.3. Rapid Scoring Functions

Different scoring functions were used to estimate protein-protein binding affinities. The procedure was to generate a set of 20 models of each complex using the homology modeling programs Modeller [41] or Rosetta [18,48], evaluate each model using each scoring function, and average the score over various subsets of the model set. The ensemble average allows one to capture the sensitivity of the scoring function to the atomistic variability of the models, and also to estimate the number of structures needed for convergence. Each structure was refined by CHARMM [42], using energy minimization. The model generation and the evaluation of the scoring functions were performed via the python program ppdx [49], which is freely available online at https://github.com/simonecnt/ppdx (accessed on 28 July 2022), and includes the scripts with parameters used to create the homology models for this study. We tested 18 scoring functions found in the literature, as described below. The first five are scoring functions developed for ranking protein-protein complexes in docking software. These are ZRANK [50] and ZRANK2 [51,52], PyDock [53], ATTRACT [54], and FireDock [55]. In addition, we tested eight statistical potentials, so called because they involve ad hoc pseudo-energy functions parametrized using structures in the PDB. Two of them, DOPE and DOPE-HR, are from the Modeller modeling suite [41,56], two are atom pair potentials developed by Rykunov and Fiser (RF_HA_SRS and RF_CB_SRS_OD) [57,58], and four are variations of the iPot potential (ipot_aace167, ipot_aace18, ipot_aace20, ipot_rrce20) [59]. Another two are commonly used energy functions, Rosetta [18] and FoldX [60,61]. Both are linear regressions of a variety of terms, including, among others, approximations to the electrostatic and dispersion binding energy, hydrogen bonds, and buried surface area. For Rosetta, we used the InterfaceAnalyzer tool [62] after relaxing the structure [63]. With FoldX we used the AnalyseComplex tool after RepairPDB, which also optimized the side chains. The final scoring function used was Prodigy [64], which is a linear regression on interchain contacts and non-interacting surface areas, fit to reproduce experimental protein-protein binding affinities. All the above scoring functions are relatively fast, with running times ranging from less than a second for the pair potentials, to several minutes for FoldX, Rosetta, or FireDock. The higher computational time for the last three methods is due to the optimization of the structure, in particular of the side-chain conformations.

### 2.4. Implicit Models of Solvation

Protein-protein binding affinities can be approximated by the interaction energy between the two proteins using implicit models of solvation [65,66]. In a popular subset of these methods, MM-GBSA, the total energy of the biomolecular system is taken to be the sum of a molecular mechanics (MM) energy, a polar solvation contribution evaluated using the generalized Born approximation (GB), and a non-polar solvation term approximated to be proportional to the solvent accessible surface area (SASA). In this model, the protein-protein binding affinity ΔG is evaluated as:

ΔG = ΔE_MM_ + ΔG_GB_ + γΔSASA, (1)

where E_MM_ is the classical energy obtained using an empirical potential energy function (here, the CHARMM36 energy function [2]), which includes bonded energy terms, and van der Waals and electrostatic interactions, G_GB_ is the generalized Born solvation free energy, and γ is an empirical surface tension coefficient. If the same structures are used for both the bound and unbound proteins, as carried out presently, the bonded interactions are the same for the bound and unbound structures, and cancel out. Two GB models were tested here: GBSW [67], and FACTS [68] as implemented in CHARMM [42]. The surface tension coefficient is an empirically tunable parameter, typically set around 0.005 kcal/mol/Å^2^ [69]. Furthermore, different implicit solvent models use slightly different definitions of the surface area, making γ non-transferable between models. Because the non-polar solvation energy is generally much smaller than the other energy terms (in view of the small surface tension coefficient above), we initially set this parameter to zero. Subsequently, to improve the agreement between the experiment and the MM-GBSA implicit solvent results, we optimized the coefficients of the individual energy terms, i.e., we wrote ΔG as:

ΔG = aΔE_elec_ + bΔE_vdw_ + cΔG_GB_ + dΔSASA + e (2)

and considered a–e to be free parameters to be fit to experimental binding affinities, as performed by others [70]. We note that the use of fitting implies that the model is not purely predictive, as it relies on parametrization using experimental data, and was included here as an example of the highest accuracy attainable with this approach.

### 2.5. Comparison between Computed and Experimental Binding Affinities

Because values computed from scoring functions are not directly interpretable as binding free energies, we quantified the agreement between the experimental binding affinities and computational results using the Pearson correlation coefficient, whose values were in the interval [−1, 1], with the left and right endpoints corresponding to perfect anticorrelation and perfect correlation, respectively. To account for the computational uncertainties in the correlations, we estimated the average and standard deviation of the Pearson coefficient using bootstrapping. Briefly, given the set of *n* (at most 20 here) computed scores for each protein-protein complex, a new set of *n* scores was sampled randomly from the original set with replacement. This random sample was averaged to obtain a value for the binding affinity prediction. The Pearson correlation coefficient was computed between this set of data and the experimental values, and the full procedure was repeated 1000 times, followed by computing the average and standard deviation.

### 2.6. An Upper Bound for the Pearson Correlation

**Figure 2:**
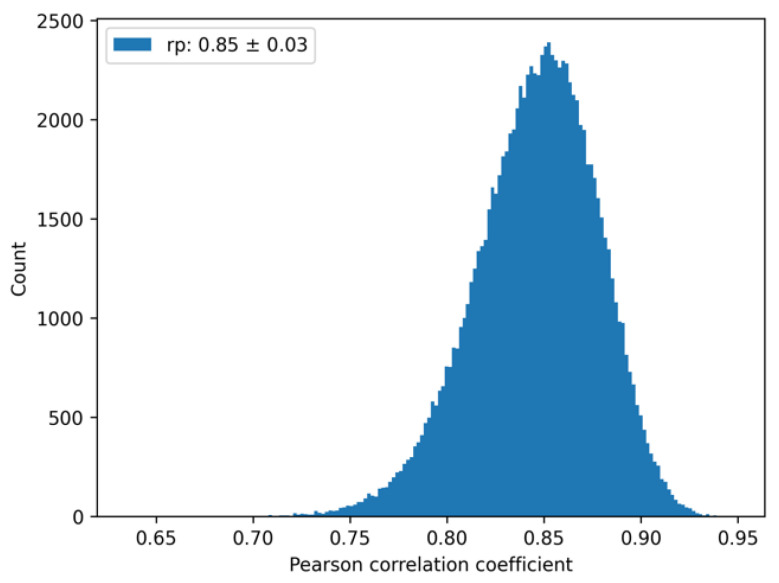
Experimental “self-correlation”. Histogram of the Pearson correlation coefficient samples from randomly generated sets. The average Pearson correlation coefficient and its standard deviation are reported in the figure legend.

We note that the presence of random error in the experiment implies an upper bound for the correlation between prediction and experiment. To account for this experimental uncertainty, we used the reported experimental random error (0.45 kcal/mol) [7], and considered each experimental value as corresponding to a Gaussian distribution with a mean equal to the experimental value, and standard deviation equal to the random error. The upper bound in the correlation can be estimated by computing correlations between different samples drawn randomly from the experimental Gaussian distributions. A histogram can be built of the distribution of the Pearson correlation coefficients, from which an average and standard deviation can be computed. Using the standard deviation of 0.45 kcal/mol for all 49 protein-protein complexes, the computed average Pearson (self-correlation) coefficient was 0.85 ± 0.03; see Figure 2. The deviation from the perfect correlation of 1 arises solely from the uncertainties in the experimental measurements. Thus, when comparing with the computations, this self-correlation value should be kept in mind as an optimal outcome.

## 3. Results

### 3.1. Binding Free Energies (bFEs) from PMF Simulations

To establish a baseline computational result, we first discuss the bFE differences associated with mutating to alanines selected residues in the Ab VRC01 bound to the gp120 antigen RSC3 [7,32]. We note that this set of mutations was simulated by Clark et al. [7], who used free energy perturbation (FEP) to alchemically transform the mutating residues in the MD simulations and obtained very good agreement with experiments. However, FEP requires a stepwise path connecting two different configurations (mutated and unmutated), but some paths are topologically very difficult to create, e.g., those for residue deletions or insertions. Because our interest is in implementing a method that can be used for affinity maturation (AM) simulations, which could, in principle, involve arbitrary, though possibly rare, sequence changes, we opted to investigate the accuracy of reversible separation of the Ab/Ag complex. Because AM can involve many mutational cycles [71], it is important to keep the simulations to a reasonable time constraint. Our present requirement was to be able to obtain a bFE value within 1–2 days, possibly using a computing cluster, on which simulations corresponding to different windows could be run in parallel.

**Figure 3:**
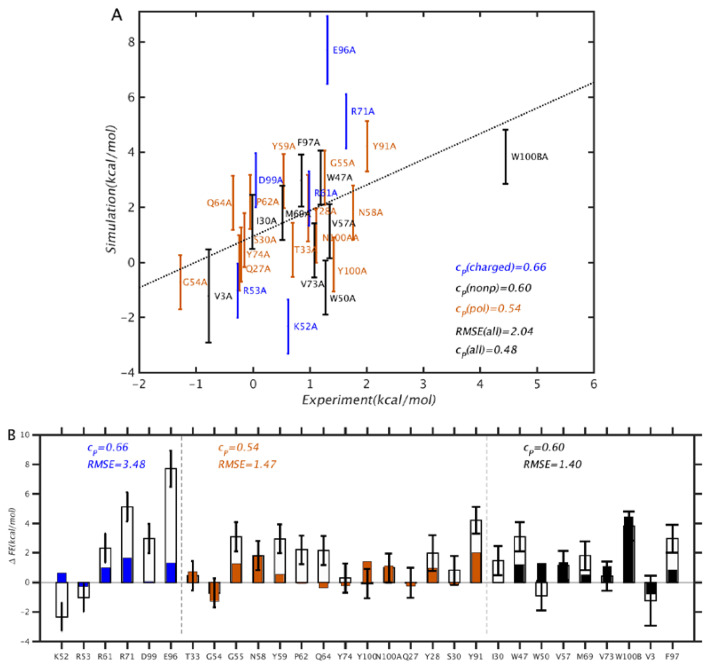
Comparison between binding free energy (bFE) differences computed from PMF simulation results and experiment. In (**A**), mutations corresponding to charged, polar, and nonpolar residues are colored in blue, brown, and black, respectively, and the dotted line corresponds to y = x. In (**B**), the mutation data are compared individually. Residues V3, Q27, Y28, S30, Y91, E96, and F97 are in the Ab light chain, and the remaining ones are in the heavy chain. The filled and unfilled bars correspond to experiment and simulation, respectively.

**Figure 4:**
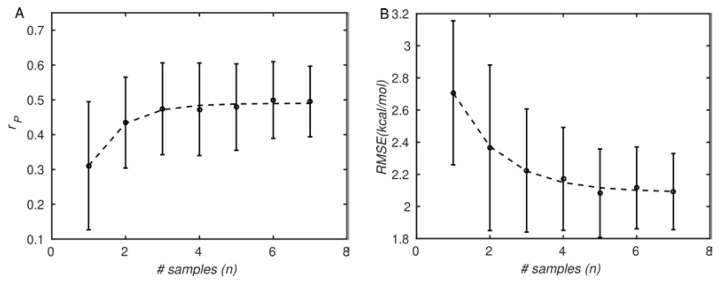
Agreement between PMF simulation results and experiments improves with the number of simulation repetitions. (**A**): Pearson correlation coefficient (*r_P_*); (**B**): root-mean-square error (RMSE). The symbols correspond to simulation and experimental data, and lines correspond to least-squares exponential fits. The error bars correspond to standard deviations, computed by bootstrapping (see Section 2. Materials and Methods).

The results of the PMF simulations are summarized in Figure 3 and Figure 4. From Figure 3, we see that the overall correlation between simulation and experiment is rather modest, at rp = 0.49 and RMSE = 2.09 kcal/mol. In particular, the discrepancies are larger than those obtained by Clark et al. [7] using free energy perturbation (FEP) combined with replica exchange with solute tempering, which were rp = 0.71 and RMSE = 0.64 kcal/mol. However, our results differ in quality depending on the type of mutation: for mutations of polar and non-polar residues, the correlation and RMSE are better, as rp = 0.56 and RMSE = 1.44 kcal/mol, whereas for charged residue mutations, the corresponding values are rp = 0.66 and RMSE = 3.5 kcal/mol. It is noteworthy that, for charged residues, rp is relatively high, and the RMSE is also much higher than average. This suggests that the simulations generally reflect the direction of the free energy change, but overestimate the magnitude, which can be explained by an incomplete conformational relaxation of the mutated residue and its environment. Further, we note that the mutations were made to alanines, which have non-polar side chains. On the basis of electrostatic considerations, we would expect mutations from non-polar residues to require less structural relaxation, and those involving charged residues to require more, which is consistent with the results in Figure 3.

As mentioned in the introduction, large biomolecules such as proteins populate many different conformations arranged hierarchically [72], the transitions between which tend to be slow and rare [29]. For this reason, using multiple structures to predict molecular properties is expected to improve accuracy, as observed by others [25,26,27,73]. This is true even for MD simulations, in which routinely affordable simulations of large systems are much shorter (10^−7^–10^−6^ s) than conformational changes (≥10^−3^ s). Therefore, in Figure 4 we examined the effect of the number of simulation replicates on the prediction accuracy. Figure 4A shows that the correlation coefficients increase from about 0.3 ± 0.2 for one sample to about 0.5 ± 0.1 for the maximum of seven samples. We note that with only three samples, the Pearson correlation coefficient is already around 0.45 ± 0.15. However, the statistical uncertainty in the correlation coefficient continues to decrease until seven samples (0.5 ± 0.1), indicating the importance of additional simulations in increasing precision. Correspondingly, the RMSE (shown in Figure 4B) decreases from about 2.75 kcal/mol to about 2.09 kcal/mol. Thus, the trends suggest that including multiple simulation replicates improves both accuracy and precision, as anticipated, although the rate of improvement is somewhat modest.

Overall, we suggest that a ballpark number of five simulation replicates be performed. Even with the seven PMF simulation replicates performed here, the uncertainties in the Pearson correlation and RMSE remain rather high. We caution, however, that our PMF method does not use enhanced sampling methods such as replica exchange or tempering [7,74]. The use of these approaches could potentially reduce the number of samples required for convergence, since they are designed specifically to accelerate exploration of configurational space, and therefore, increase decorrelation within single simulation trajectories.

### 3.2. Scoring Functions

**Figure 5:**
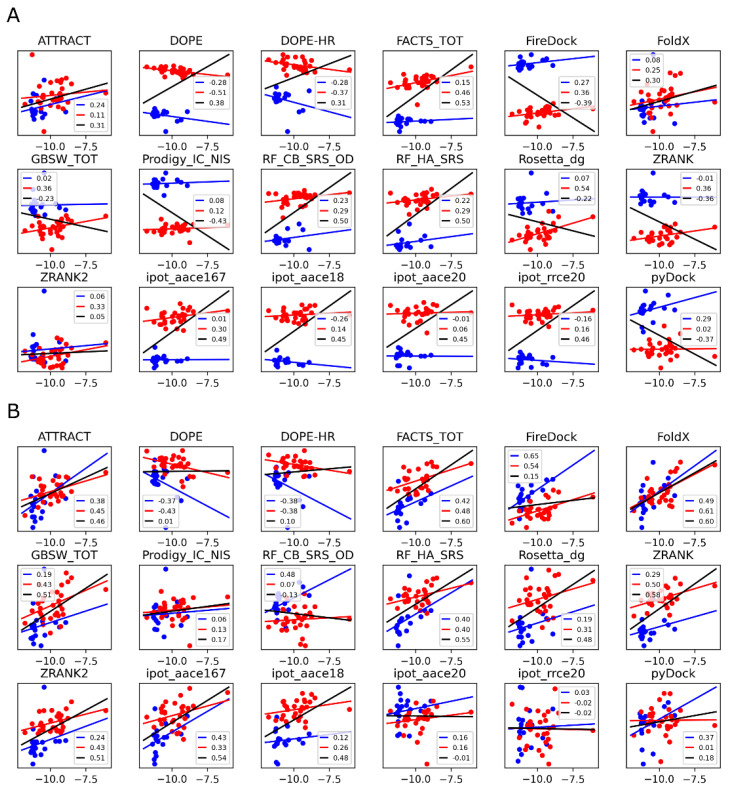
Correlations between the experimental and computed binding affinities using the 18 quick scoring functions. (**A**): the structures were generated using Modeller; (**B**): the structures were generated using Rosetta. Data for the complexes with the RSC3 antigen are in red, data for the complexes with the 93TH057 antigen are in blue, and the black lines correspond to the overall correlations. The numbers in the legends are the Pearson correlation coefficients.

**Figure 6:**
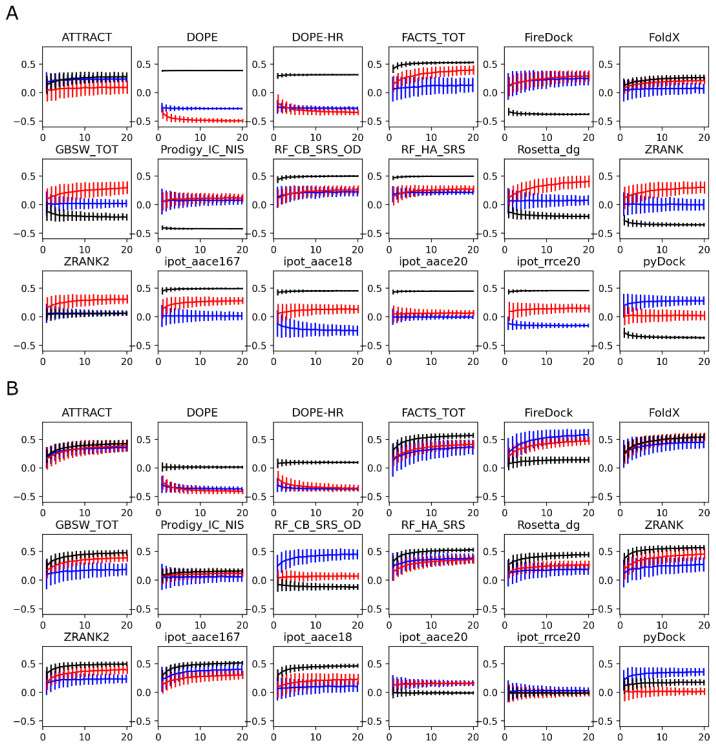
Convergence of the Pearson correlation coefficients between experimental and computed binding affinities with the number of models used in the average for each scoring function. (**A**): the structures were generated using Modeller; (**B**): the structures were generated using Rosetta. Data for the complexes with the RSC3 antigen are in red, data for the complexes with the 93TH057 antigen are in blue, and the black lines correspond to the overall correlations.

Given the high computational expense and the modest result quality of the above PMF simulations, it is instructive to consider whether scoring functions could provide a similar level of accuracy, but at a significantly reduced computational cost. The results of the 18 scoring functions considered here (see Materials and Methods) are summarized in Figure 5 and Figure 6. The data corresponding to the RSC3 and 93TH057 antigens are plotted separately, shown as red and blue linear regression fits, respectively, with the overall regression fit shown in black. Results in panels A and B of the figures correspond to model structures generated using Modeller [41,56] and Rosetta [18], respectively.

In the case of Modeller-generated structures, most of the scoring functions show a systematic shift that depends on the antigen present in the protein-protein complex (the red and blue data series in Figure 5A). If each of these two series are analyzed separately (the red and blue regression lines), a weak positive correlation is generally observed, and in some cases, a negative (anti-)correlation. Because of the antigen-dependent systematic shift mentioned above, treating the two datasets as one (the black regression line) is misleading. For example, the DOPE-HR scoring function shows a significant anticorrelation when the two series are analyzed separately, but the overall correlation is positive. The opposite is true for pyDock, with positive correlations observed for the two sets if considered separately, but an anticorrelation observed if the data are merged. Only three scoring functions do not show this problem with the Modeller structures: ATTRACT, FoldX, and ZRANK2. In these scoring functions, the separated or merged correlations are similar and consistent, but still low (Pearson coefficients of 0.3 or less).

Considering the results of the same scoring functions applied to the structures generated by Rosetta (Figure 5B), the antigen dependence is much reduced. The overall correlations are also much more in line with the correlations of the two series analyzed separately. The best performing scoring functions are ATTRACT (rp = 0.46), FoldX (rp = 0.60), RF_HA_SRS (rp = 0.55), and ipot_aace167 (rp = 0.54). The implicit solvent models FACTS_TOT and GBSW_TOT also perform well (overall rp of 0.60 and 0.51, respectively).

In Figure 6, in which panels A and B are the structures generated using Modeller and Rosetta, respectively, we show the evolution of the correlation coefficients as a function of the number of structures used to compute the average score of each protein-protein complex, up to the maximum number 20. For the Modeller structures (Figure 6A), the red and blue curves corresponding to the different antigens are generally similar, whereas the black curve is very different; this discrepancy is due to the systematic antigen-dependent shifts observed in Figure 5A. In Figure 6A, one of the best scoring functions is FoldX. In the corresponding plot, all three curves (red, blue, and black) are similar, but the overall correlation coefficient at 20 structures is low, at about 0.3. Despite the low final value, it is noteworthy that the correlation coefficient improves significantly when more than one structure is considered, similar to what was found for the PMF simulations. Specifically, the correlation coefficient increases monotonically with the number of structures considered. A similar trend is observed for all of the scoring functions.

For the Rosetta-modeled structures (Figure 6B), as observed before in Figure 5B, the systematic shift is less pronounced, which is reflected in smaller differences between the three correlation curves. Further, the correlation coefficients are generally higher, reaching 0.6 for several scoring functions. The increase in the correlation coefficient with the number of structures is more pronounced for the best-performing scoring functions, such as ATTRACT or FoldX. About 10 structures appear to be needed to reach 90% of the correlation obtained with the 20-structure ensemble average, with plateaus not fully reached even for the 20 structures. A factor that may play a role in the number of structures needed to reach convergence is the diversity of the generated conformational ensemble. To quantify the diversity of the structures used here, we computed the pairwise heavy-atom RMSDs between the 20 models generated for each complex (see Figure S1). We found that Modeller generally creates more similar structures, compared to Rosetta, which results in lower RMSD values. Higher variability is observed for the complexes involving the RCS3 antigen, which is possibly due to the lower sequence similarity to the template (template structure of the complex contains the 93TH057 antigen).

### 3.3. MM-GBSA with Optimized Coefficients

**Figure 7:**
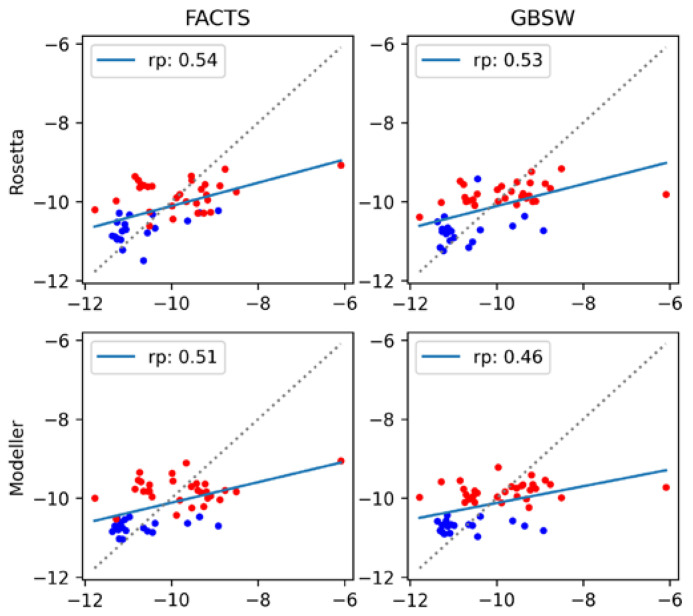
Correlation between experimental and computed binding affinities using the refitted MM-GBSA models. FACTS and GBSW implicit solvents correspond to the (**left)** and (**right)** columns, respectively; Rosetta and Modeller structures correspond to the (**top)** and (**bottom)** rows, respectively.

The final set of results is obtained from MM-GBSA implicit solvent models, in which the contributions of the constituent energy terms are adjusted to maximize agreement with the experiment. As described in the Materials and Methods section, the binding energy is the sum of the electrostatic and van der Waals energies, a generalized Born (GB) solvation energy and a nonpolar solvation contribution taken to be proportional to the solvent accessible surface area (SASA) [69]. Because the energy terms in MM-GBSA are grounded in physics, the weight of each term should be unity. However, in practice, the weights can be tuned to improve fit to the experiment (Equation (2)). This fitting procedure was performed using two GB implicit solvent models, FACTS and GBSW, for structures generated by both Modeller and Rosetta, and the results are summarized in Figure 7 and Table 2. First, we note from Figure 5 that, without fitting of the weights, FACTS and GBSW behave similarly to the other scoring functions (see in Figure 5 the plots labeled FACTS_TOT and GBSW_TOT). The correlation coefficients exhibit the same antigen-dependent shifts as observed for the other scoring functions, the effect being less pronounced for Rosetta-generated vs. Modeller-generated structures.

The coefficient optimization generally improves agreement with the experiment, especially for the structural ensemble generated by Modeller (compare Figure 7 with Figure 5). The system dependence is less pronounced, and the correlation coefficients are comparable to the best scoring functions (rp = 0.54 for the refit FACTS model applied to Rosetta structures). It is noteworthy that the predictions from the refitted models are not qualitatively better for Rosetta-generated structures than for Modeller-generated structures. In essence, the fitting procedure is able to reduce the dependence on the type of structural modeling used.

An apparent advantage of the refitting procedure is that the resulting energies are better comparable to the experimental values, with the RMSE values of 0.88–0.92 kcal/mol (Table 2). However, it should be emphasized that the energy term coefficients in Table 2 are difficult to justify on physical grounds, with some of them, in particular those of the GBSW model, taking on negative values. For example, examination of the energy contribution coefficients in Table 2 shows that, for the Modeller structures scored with the FACTS GB model, the van der Waals (vdW) term is attenuated by several-fold relative to the electrostatic and GB terms, whereas for the Rosetta-generated model structures, the vdW term is actually higher. Thus, although by using the fitting procedure we improve agreement with the experiment, we also forgot the physicality of the model.

## 4. Concluding Discussion

Efficient and accurate calculation of binding free energies (bFEs) between proteins is an essential component of antibody (Ab) and antigen (Ag) design. In this study, we compared several methods of computing bFEs in an effort to find an optimal compromise in speed and accuracy to use in affinity maturation simulations, and in antibody and antigen design. The methods considered the range from computationally expensive MD simulations to compute potentials of mean force (PMFs), which required 1–2 days to obtain a single value, to empirical methods that use fast but highly approximate scoring functions to produce values within seconds to minutes. For the methods in the second category, the cost of modeling mutant structures using sophisticated homology building tools [16,18] can be dominant.

Our results from the expensive all-atom PMF simulations have reasonable accuracy when tested on a small set of alanine mutations [7]. However, we were able to obtain results of similar accuracy using scoring functions. We note that results of higher accuracy were reported using free energy perturbation (FEP) simulations for the same dataset [7,14], which suggests that FEP may be the method of choice for relatively simple structural mutations that can be easily represented by the dual-topology alchemical paradigm. On the other hand, the PMF method could be still preferable in cases that involve topological changes to the protein backbone, such as deletions and insertions of one or more residues, that are currently too complicated to incorporate into a dual-topology paradigm required by modern alchemical algorithms. We caution that the present test set of simple alanine-scanning mutations may not represent the accuracy of the method when it is applied to such complicated mutations.

In addition to comparing different methods, we considered the effect of the number of independent structures (for rapid scoring methods) or the number of independent simulation replicates (for PMF simulations) on the prediction accuracy. In broad agreement with previous results from other groups [26,27], we observed an increase in accuracy with the number of structures, but the rate of increase was dependent on the method used. Our recommendation based on the present results is to use about ten structures for scoring function methods, and about five simulation replicates for MD-based methods, but the precise number will depend on the method details, such as the protocols for structure modeling. In the case of limited experimental structures (as is the case of the single X-ray template structure used here), additional structures could be generated using homology modeling software, or from MD simulations. Alternatively, algorithms for incorporating conformational flexibility via backbone and side-chain coordinate sampling can be employed [25,73,75,76,77]. However, even with additional sampling, it can be challenging to adequately capture the conformational ensembles of antibody CDR regions, which can use flexibility to achieve not only specificity, but also breadth [78]. Another limitation of the present homology-model-based approach to computing binding affinity changes is that the use of a small number of template structures prevents significant reorientation of the bound antibody. Thus, the structure generation protocols used here may be inaccurate for modeling maturation of antibodies that reorient upon maturation. Use of docking software such as HADDOCK [79] as an additional structure refinement step may be beneficial in such cases.

As a further step to improve accuracy, we considered empirically tunable binding affinity methods, trained with some pre-existing binding data. In particular, we used an energy decomposition formalism similar to that used in molecular mechanics-generalized Born surface area (MM-GBSA) models to optimize the additive contributions of the different energy terms to bFE, as carried out by others [70]. Although this approach yielded the best agreement with the experimental binding free energy differences, as is to be expected in view of the regression procedure, it suffers from the drawback of requiring a “training” set, and is therefore not *a priori* predictive. In addition, because the energy contribution coefficients were allowed to vary arbitrarily, so as to obtain an optimal fit, some of them turned negative (see Table 2), clearly demonstrating unphysical behavior. Nevertheless, such a semi-empirical approach may be justified if even a modest number of experimental binding values can be obtained for model training. A further extension of the fitting procedure could be to expand beyond linear regression to more complex machine learning methods [80,81].

A final practical note is that, in view of the limited accuracy reported in our study, there is a high probability that computational optimization using the binding affinity methods described here will not lead to improved affinity for any particular antigen or antibody sequence. However, highly optimized antibodies can still be discovered despite the rather modest correlations provided the optimization strategy is used to create a large, preferably diverse, sequence ensemble, rather than a small number of mutants.

## Figures and Tables

**Table 1:** Dataset of the 49 experimental binding affinities used in this work, adapted from Refs. [7,32]. Energies are expressed in units of kcal/mol. The prefix H- or L- indicates whether the mutation was made to the heavy or the light chain, respectively; letter pairs at the end of some of the mutants correspond to the residue insertion code relative to the germline sequence, followed by the 1-letter code of the residue after mutation. The reversion mutant definitions are taken from Table S10a in Ref. [32]. They are: H-4rev—A56G, V57T, P62K, V73T (heavy chain); H-7rev—T33Y, G55S, A56G, V57T, P62K, V73T, Y74S (heavy chain); HL-11rev chain: I30T, K52N, R53N, G54S, A56G, V57T, R61Q, P62K, V73T, Y74S (heavy chain), Y28S (light chain).

93TH057/VRC01		RSC3/VRC01	
Mutation	ΔG	Mutation	ΔG
-	−11.31	-	−10.51
H-T33Y	−11.23	H-I30A	−10.51
H-G54S	−10.56	H-T33A	−9.81
H-A56G	−11.37	H-W47A	−9.32
H-V57T	−11.17	H-W50A	−9.23
H-P62K	−11.08	H-K52A	−9.89
H-R61Q	−11.21	H-R53A	−10.77
H-K52N	−11.13	H-G54A	−11.78
H-R53N	−11.26	H-G55A	−9.26
H-V73T	−11.14	H-V57A	−9.17
H-Y74S	−10.98	H-N58A	−8.76
H-I30T	−11.26	H-Y59A	−9.97
H-4rev	−9.63	H-R61A	−9.53
H-7rev	−9.36	H-P62A	−10.55
HL-11rev	−10.44	H-Q64A	−10.85
L-Y28S	−10.65	H-M69A	−9.99
H-C32S+H-C98A	−11.05	H-R71A	−8.88
L-iAA	−8.92	H-V73A	−9.43
L-iSY	−10.38	H-Y74A	−10.71
		H-D99A	−10.45
		H-Y100A	−9.10
		H-N100AA	−9.40
		H-W100BA	−6.09
		L-V3A	−11.28
		L-Q27A	−10.74
		L-Y28A	−9.54
		L-S30A	−10.65
		L-Y91A	−8.51
		L-E96A	−9.20
		L-F97A	−9.66

**Table 2:** Fitted parameters (Equation (2)) and regression statistics for the four models generated by the GBSW and FACTS implicit solvents on the Rosetta and Modeller structures. The statistical uncertainty in the Pearson correlation coefficients is about 0.02, estimated from the leave-one-out error. Energies are expressed in units of kcal/mol.

	Rosetta		Modeller	
	FACTS	GBSW	FACTS	GBSW
a (E_elec_)	0.0478	−0.0157	0.0225	−0.0111
b (E_vdw_)	0.0961	−0.0651	0.0036	−0.0687
c (E_GB_)	0.0541	−0.0131	0.0307	−0.0053
d (SASA)	0.1375	0.3614	0.2736	0.1618
e	−3.65	−5.39	−4.77	−13.60
Pearson:	0.54	0.53	0.51	0.46
Slope:	0.29	0.28	0.26	0.21
Intercept:	−7.17	−7.32	−7.52	−8.01
*p*-value:	5.63 × 10^−5^	9.48 × 10^−5^	1.85 × 10^−4^	8.79 × 10^−4^
RMSE:	0.87	0.88	0.89	0.92

## Data Availability

Data are contained within the article or supplementary materials. The model generation and the evaluation of the scoring functions were performed via the python program ppdx, which is freely available online at https://github.com/simonecnt/ppdx (accessed on 28 July 2022). MD simulation trajectories from our study can be found as an online dataset at https://zenodo.org/record/6879091 (accessed on 28 July 2022).

## Supplementary Materials

The following supporting information can be downloaded at: https://www.mdpi.com/article/10.3390/antib11030051/s1, Figure S1: Pairwise RMSDs between models. For each mutated complex, the pairwise heavy-atom RMSDs between 20 generated models were computed; the average, minimum, and maximum RMSD values are plotted (diamond, lower bar, and upper bar, respectively). The points on the X-axis are ordered as in Table 1 (main text). Top: structures were generated using Modeller; bottom: structures were generated using Rosetta.
