# Supplementary material for: On the Rapid Calculation of Binding Affinities for Antigen and Antibody Design and Affinity Maturation Simulations
Source: Antibodies (Basel). 2022 Aug 3;11(3):51. doi: 10.3390/antib11030051 (PMC9397028; doi:10.3390/antib11030051)
Supplement: Supplementary file 1 [file antibodies-11-00051-s001.zip › antibodies-1816607-supplementary.pdf]

Supplementary material

On the Rapid Calculation of Binding Affinities for Antigen and Antibody Design and Affinity Maturation Simulations

Simone Conti[1*], Edmond Y. Lau[2], Victor Ovchinnikov[1]

[1] Department of Chemistry and Chemical Biology, Harvard University, Cambridge, MA 02138, USA
[2] Physical and Life Sciences Directorate, Lawrence Livermore National Laboratory, Livermore, CA, 94551
* Correspondence: simonecnt@gmail.com

Supporting Information

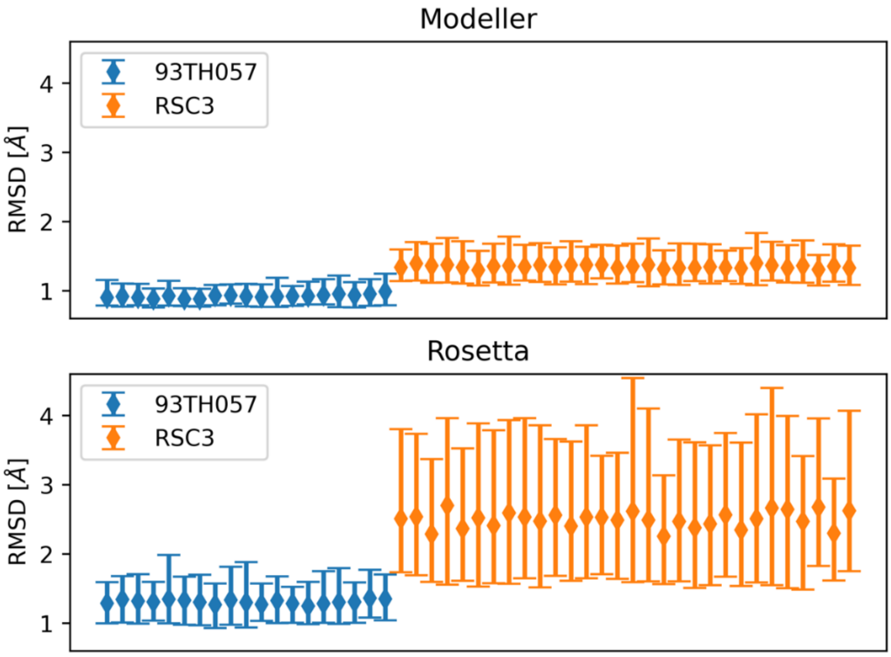

Figure S1. Pairwise RMSDs between models. For each mutated complex, the pairwise heavy-atom RMSDs between 20 generated models were computed; the average, minimum, and maximum RMSD values are plotted (diamond, lower bar, and upper bar, respectively). The points on the X-axis are ordered as in Table 1 (main text). Top: structures were generated using Modeller; bottom: structures were generated using Rosetta.

Modeller generally creates more similar structures compared to Rosetta, which results in lower RMSD values. Higher variability is observed for the complexes involving the RCS3 antigen, which is possibly due to the lower sequence similarity to the template (template structure of the complex contains the 93TH057 antigen).
